# Institutionalizing research capacity strengthening in LMICs: A systematic review and meta-synthesis

**DOI:** 10.12688/aasopenres.13116.3

**Published:** 2021-02-12

**Authors:** Marta Vicente-Crespo, Ojo Agunbiade, John Eyers, Margaret Thorogood, Sharon Fonn

**Affiliations:** 1 African Population and Health Research Center, African Population and Health Research Center Campus, Nairobi, Kenya; 2 School of Public Health, University of the Witwatersrand, Johannesburg, South Africa; 3 Sociology and Anthropology, Obafemi Awolowo University, Ile Ife, Osun State, Nigeria; 4 International Initiative for Impact Evaluation, London, UK; 5 The University of Warwick, Coventry, UK

**Keywords:** institutional research capacity strengthening, higher education, capacity development, institutionalization

## Abstract

**Background**: Evidence on effective strategies to ensure sustainability of research capacity strengthening interventions in low- and middle-income country (LMIC) institutions is lacking. This systematic review identified publications describing research capacity building programs and noted their effect, their contexts, and the mechanisms, processes and social actors employed in them.

**Methods**: We searched online databases for the period 2011-2018. Inclusion criteria were that the publications 1) described the intervention; 2) were implemented in LMICs; 3) were based in, or relevant to, university staff or post docs; 4) aimed to improve research capacity; 5) aimed to effect change at the institutional level. Two reviewers screened titles, abstracts and full text in consecutive rounds, a third resolved disagreements. Two people extracted the data of each full text using a data extraction tool covering data relevant to our question.

**Results**: In total 4052 citations were identified and 19 papers were included, which referred to 14 interventions. Only three interventions mentioned using a conceptual framework to develop their approach and none described using a theory of change to assess outcomes. The most frequent inputs described were some method of formal training, promotion of a research-conducive environment and establishment of research support systems. A range of outcomes were reported, most frequently an increased number of publications and proportion of staff with PhDs. When factors of success were discussed, this was attributed to a rigorous approach to implementation, adequate funding, and local buy-in. Those who mentioned sustainability linked it to availability of funds and local buy-in. The lack of a common lexicon and a framework against which to report outcomes made comparison between initiatives difficult.

**Conclusions**: The reduced number of interventions that met the inclusion criteria suggests that programs should be well-described, evaluated systematically, and findings published so that the research capacity strengthening community can extract important lessons.

## Introduction

There has been a growth in investment for research capacity development in institutions in low- and middle-income countries (LMICs). However, the long-term success and sustainability of increased research capacity depend on the degree to which successful interventions are mainstreamed within the routine functioning of institutions and the research-system. Increasing the number of African PhD-level graduates is essential but not sufficient for sustainability; these researchers have to be both research active in Africa and build the next generation of researchers. To achieve this, they need research supportive environments, and this requires intuitional or system-level change in higher education institutions ^1^. Institutionalisation of research capacity strengthening is multifaceted, complex and requires buy-in at both the individual and system level ^2– 4^.

The Consortium for Advanced Research Training in Africa (CARTA) comprises eight African Universities and four African research centres across eight countries with a range of non-African partners ^5^. It was set up to build research capacity in African universities, including interventions to inspire and promote institutional change. It comprises a compendium of interventions including some system-level interventions to promote institutional change, such as internationally competitive PhD training for existing university staff, improved research supervision through PhD supervisor workshops, a range of interventions with relevant academic and administrative staff at universities to promote research supportive environments, and support to CARTA graduates to enable them to remain research-active and located in Africa ^1^. CARTA has an overt ‘institutionalisation’ strategy, which seeks to ensure that CARTA interventions that are valued by member universities become formally embedded in the functioning of those universities. CARTA has invested in strategies to institutionalise CARTA innovations into routine university systems, but the most effective strategies to achieve this are not obvious. Here we report our findings from a systematic review carried out to identify effective strategies for institutional-level research strengthening within universities in LMICs with the aim of informing our own approach. In particular, we were interested to know which strategies have been effective elsewhere and the contexts, mechanisms, processes and social actors that aided the institutional change achieved.

## Methods

We carried out a systematic review and narrative synthesis of interventions aiming at institutional research capacity strengthening in LMICs. The criteria for the review reflects its purpose ( Box 1). For an article to be included, it should describe the intervention carried out beyond the description of how the trainees, fellows or participants were selected. The interventions described must be implemented in LMICs, be based in universities and aim at improving research capacity strengthening at a level beyond the individual.

**Box 1. Inclusion criteria**

1. Describes an intervention (articles describing only selection of recipients are excluded)
2. Intervention is implemented in LMIC
3. Intervention based in, or relevant to, university staff or post docs
4. Intervention aims to improve research capacity
5. Intervention aims for change at the group/institutional/systematic/systemic level (intervention addressing only individuals are excluded)

### Database searches

Searches were run in a number of subject-specific and multidisciplinary bibliographic databases for the period 2011–2016 with a search strategy using both natural language (title/abstract) and controlled language (database index terms) strings adapted for each database (See Supplementary Table 1, *Extended data* ^6^). The searches were last performed on 14th August 2018. A search filter for LMICs (adapted from a Cochrane EPOC group filter based on World Bank definitions) was used to restrict papers. The following databases were searched: Medline (Ovid), Embase (Ovid), ERIC (education database - Ebsco), Scopus, International Bibliography of the Social Sciences (Proquest), Public Affairs Information Service (Proquest), Social Science Journals (Proquest), Higher Education Empirical Research (HEER - up to 2014). Results were amalgamated within Endnote X6.0.1 and duplicates removed, making a total of 2431 citations for screening. This search was further updated with the same databases (except HEER) to August 2018 and this retrieved a further 1503 citations after duplicates were removed. No language limits were placed on the searches.

### Other strategies to find relevant papers

Once we had the included studies from the initial searches, we used them to extend our search. We manually screened through the references of all included papers, we searched for other articles describing the included interventions, and we performed a citation search of the included articles in Scopus, retrieving a further 89 potentially relevant articles. We also looked at the references included in a narrative review of overlapping interests published in 2019 ^4^.

### Selection of papers

Text files ( *.txt*) exported from EndNote X6.0.1 were converted to . *bib* format using Mendeley1.19.4 and then to . *csv* using Jabref 5.0. The titles obtained in the two searches were selected in a similar manner. For the 2011–2016 search, we first reviewed the titles and selected those that were possibly relevant, always choosing to include rather than exclude any title where there was doubt. Each title was reviewed by one person. For the search up to 2018, two people independently read through the titles and any conflicting decisions resolved by a third team member. At the next stage, all selected titles and the relevant abstracts were reviewed independently by two people and any conflicting decisions resolved by a third team member. Next, the full text of the papers was obtained and again reviewed independently by two people, with a third resolving any conflicts.

### Data extraction

To develop a data extraction form, four reviewers read the same two papers, noting what information should be extracted, and then met to develop a data extraction form in Redcap 9.1 - 9.3 ^7, 8^. Using the online form, data was again extracted by two members of the team independently and conflicts resolved by a third person. The extraction tool allowed us to extract information on the country and institution where the intervention was implemented, the initiator of the intervention, the various inputs included in the intervention, the reported outcomes, and whether there was a direct reference to sustainability.

The full papers were read by two people who checked for inclusion and extracted the data. Conflicts on inclusion were resolved by a third reviewer. Extracted data was merged with three people going over the available extracted data and referring to the full article in case of conflicting information coming from the two extractions.

### Quality appraisal

The reviewers extracting the information from each article assigned them a score between 1 and 5 depending on the degree to which the data we were looking for was included in the paper ( Box 1). The scores were averaged.

## Results

**Figure 1.**
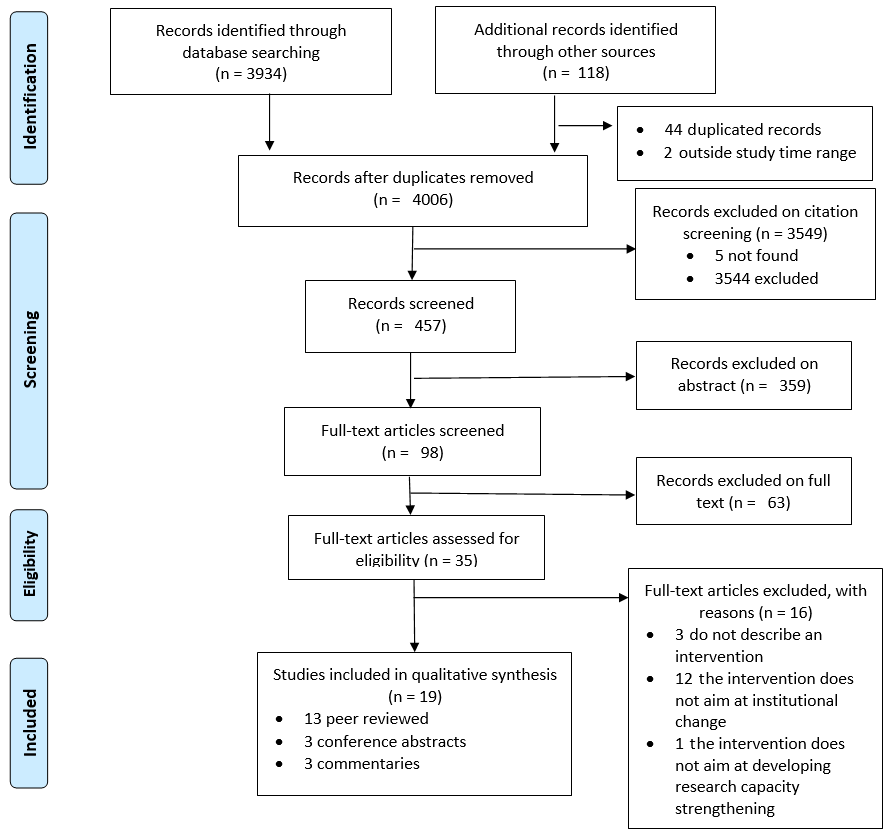
PRISMA flow diagram of the study selection process (Modified from 9 under a CC BY 4.0 license).

Our initial searches identified 3934 citations. Through other searches, we identified 118 articles, of which 44 were duplicates from the first search, and two were papers published in 2019, outside of our study time range. This left 4006 citations to screen, from which we selected 462 as possibly relevant. There were five citations which we were unable to trace. We checked the abstract (or the full paper if that was all that was available) for the remaining 457 and selected 98 papers. Of these, 63 were excluded after a second reading, and 35 were selected for data extraction. A further 16 were excluded at the stage of data extraction, mostly because the intervention described did not aim at institutional change or did not contain a description of an intervention ( Figure 1). Of the 19 papers finally included (Supplementary Table 2, *Extended data* ^6^), 13 articles came from the initial systematic search, one paper was found by checking the references of included papers, another paper was found looking for other articles describing included interventions, and four papers were found through the citation search.

We included 19 papers relating to 14 interventions or groups of interventions. For two interventions (SJTU ^10^, Sacore ^11^), we found only a commentary, and for another two (PRIME-K ^12^, KATH ^13^) only a conference abstract. In one case (UHon ^14, 15^), we found both a conference abstract and full paper. For seven interventions we found one full article (IDIMak ^16^, TDR ^17^, CoMM ^18^, WCape ^19^, Lmpp ^20^, ARCADE ^21^, WHSRO ^22^), for one intervention we found two articles (CHSZim ^23, 24^) and for one intervention we found three articles and a conference abstract (MozC ^25– 28^). We contacted the authors of two papers for further clarification and received a reply from both. Details of the included papers are shown in Table 1.

**Table 1.** Characteristics of included papers by intervention.

A	B	C	D	E	F	G
Brief name of intervention and number of papers	Peer reviewed paper (Y/N)	Country(ies) of implementation	1st author from country of implementation Y/N	Institution	Initiator of intervention (the 'driving force')	Quality score: 0-5 (best)
IDIMak	Y	Uganda	Y	U Makerere	Internal	3.3
TDR	Y	Many countries, not specified	N	Many institutions, not specified	TDR	2.8
CoMM	Y	Malawi	Y	College of Medicine, U Malawi	Internal	2.2
KATH	N	Ghana	Y	Komfo Anokye Teaching Hosp	Internal	2.5
MozC ^1^	y	Mozambique	Y	Universidade Eduoardo Mondalane, Unilurio, Unizambeze	Mozambican partners	1.7
MozC ^2^	N	As above	Y	As above	As above	4.2
MozC ^3^	Y	As above	Y	As above	As above	4.8
MozC ^4^	Y	As above	Y	As above	As above	4
UHon ^1^	Y	Honduras	N	U of Honduras	International partnership	0.7
UHon ^2^	Y	As above	N	As above	As above	5.0
CHSZim ^1^	Y	Zimbabwe	Y	U Zimbabwe College of Health Sciences	International partnership	3
CHSZim ^2^	Y	As above	Y	As above	As above	4.6
SJTU	N	China	Y	Shanghai Jiao Tong U	Internal	2.0
Sacore	N	Malawi, Zambia, Zimbabwe	Y	College Medicine U Malawi, School Medicine, U Zambia, College Health Sciences, U Zimbabwe	Not clear	2
Lmpp	Y	South Africa	Y	U Limpopo	Internal	3.9
PRIME-K	N	Kenya	Y	U Nairobi	International partnership	0.9
ARCADE	Y	Africa & Asia	N	Many institutions, not specified	International partnership	1.9
WCape	Y	South Africa	Y	U Western Cape	Internal	4.4
WHSRO	Y	South Africa	Y	Health Science Research Office, U Witwatersrand	Internal	4

Two papers reported multiple interventions with multiple partners. One was authored by members of the funding institution (TDR) and the other reported on two large EU Framework funded collaborations, one in Africa and one in Asia (ARCADE). The other 17 papers concerned single-institution interventions, although three of them were funded through a single initiative; the US President’s Emergency Plan for AIDS Relief (PEPFAR) Medical Education Partnership Initiative (MEPI). One intervention was based in Latin America (UHon), one in China (SJTU) and the remaining eight in sub-Saharan Africa. All the interventions focused on health sciences, although this was not a criterion of our search. In all but three papers (UHon, Arcade, TDR) the first author was from an institution in the country where the intervention(s) occurred. Three of the interventions appeared to have been conducted without any external funding (Lmpp, SJTU, WHSRO). In these cases, the intervention was responding to national within-country higher education system incentives. All the other interventions relied on funding from Northern institutions of some kind.

There was little description of how the interventions were designed, and only three interventions mentioned using a conceptual framework to develop the capacity building approach (Lmpp, IDIMak, WCape). All the three frameworks were specifically developed for use in LMICs but differed in their approach ^29– 31^. None of the sets of authors commented on the value of the frameworks they had used. None of the 14 interventions described using any theory of change to assess outcomes.

We classified the various inputs that the 14 interventions employed into five categories ( Table 2). There were 12 interventions that offered some form of *training* which included: formal masters’ degree or diplomas in various subjects, non-for-credit courses, online or blended learning; short courses; writing skills training for publication or grants. Seven developed *research support systems* which offer grants management, data management and analysis, library access, academic support, etc. There were nine interventions which we summarised as promoting a *conducive research environment*. This included initiatives such as providing paid time off to focus on research, mentorship, visiting scholars, setting up research groups and facilitating collaborative research. Three institutions provided *personal incentives* to individuals who produced research outputs or supervised successful students (see Supplementary Table 3, *Extended data* ^6^). In all cases, there was a monetary reward, and one also included tenure. Five institutions also reported inputs in *infrastructure*.

**Table 2.** Reported inputs employed by each of the 15 interventions to achieve change. *(See text for a description of the categories)*.

Short name	Training	Research support systems	Conducive environment	Incentives	Infrastructure improved
IDIMak	Yes	Yes	Yes	No	No
TDR	No	No	Yes	No	Yes
CoMM	Yes	Yes	No	No	No
KATH	Yes	Yes	No	No	No
MozC	Yes	Yes	No	No	Yes
UHon	Yes	No	Yes	No	Yes
CHSZim	Yes	Yes	Yes	No	Yes
SJTU	No	No	Yes	Yes	No
SACORE	Yes	Yes	Yes	No	No
Lmpp	Yes	No	Yes	Yes	No
PRIME-K	Yes	Yes	Yes	No	No
ARCADE	Yes	No	No	No	No
WCape	Yes	No	No	No	No
WHSRO	Yes	No	Yes	Yes	Yes
TOTAL	12	7	9	3	5

We similarly classified the various reported outputs into 11 categories. Nine interventions reported increased publications, six reported increases in the proportion of staff with PhDs, and five interventions reported new infrastructure, new partnerships, increases in research income, or research supportive policies ( Table 3). Other interventions mentioned included, for example, that staff retention improved and that expatriates returned (CoMM) (Supplementary Table 3, *Extended data* ^6^). 

**Table 3.** Summary of reported outcomes of the interventions.

Short name	Publications increased	Grants writing	Research income	Research support policies	New infrastructure	New partnerships	Increase in staff PhDs	University ranking	New research unit	Staff retention and expats returning	Conference organised
IDIMak	Yes										
TDR					Yes		Yes				
CoMM			Yes	Yes						Yes	
KATH	Yes	Yes		Yes							
MozC	Yes	Yes	Yes	Yes	Yes	Yes					
UHon	Yes			Yes	Yes	Yes					Yes
CHSZim	Yes		Yes		Yes	Yes				Yes	
SJTU			Yes				Yes	Yes			
SACORE	Yes			Yes			Yes				
Lmpp	Yes						Yes				
PRIME-K						Yes					
ARCADE		Yes	Yes			Yes					
WCape	Yes						Yes		Yes		Yes
WHSRO	Yes				Yes		Yes				
	9	3	5	5	5	5	6	1	1	2	2

We looked for what authors thought might account for the success of their intervention and, separately, whether they thought their intervention was sustainable and, if so, what might account for sustainability. In the six interventions which discussed factors affecting successful outcomes, success was variously attributed to a rigorous approach to implementation (KATH, WCape), generous funding (IDIMak), local buy-in (CoMM, MozC), and implementing a wide range of interventions to meet different needs (WHSRO). The rest of the reports did not include any comment on factors affecting success.

Of the six interventions where authors considered sustainability, five were considered to be sustainable (SJTU, KATH, MozC, CoMM, WCape). The intervention in China (SJTU) relied on government funding, which was continuing, and this was also the case with the intervention in the Western Cape of South Africa (WCape). The group implementing the intervention in Mozambique (MozC) thought that including the heads of the three universities together with government ministries from the beginning was important in ensuring sustainability. Similarly, the group from Malawi (CoMM) thought that local buy-in was important. The group from Ghana (KATH) described an intervention that had been underway for 10 years and no longer required external funding. The group from Limpopo, South Africa (LMPP) were pessimistic about sustainability since there was an imminent amalgamation with another higher education institution. 

## Discussion

In this systematic review, we aimed to identify effective strategies to incorporate research capacity building activity into the routine functioning of institutions, thus ensuring long term sustainability. We looked for papers which described interventions that focused on research capacity and targeted a group/institutional/system level. Disappointingly, all the interventions we found reported results in a non-systematic manner, making it impossible to draw comparisons. Moreover, outcomes were reported without connection to the inputs, making it difficult to identify any direct contribution. We were surprised that we did not find any interventions that focused on capacity development beyond the health sciences, as interventions to build research capacity in agriculture and economics are well known and long-standing ^32, 33^.

In our approach, we hypothesised that what people did, with whom, and how, will affect both the immediate outcome and longer-term sustainability. To develop effective interventions, it is important to start with a theory of change or a conceptual framework ^34^. Three papers mentioned a framework, but none of the papers articulated a hypothesis of how the desired outcomes were related to the various parts of the intervention, that is, there was no explicit theory of change. This is required so that relevant elements of the intervention, as well as outcome and output indicators, can be defined. We are not alone in noting that theories of change are not always articulated or if they are that they are not clearly linked to the indicators that are reported ^3^.

None of the papers described interventions that the authors considered to be failures; all the outcomes reported were seen as successes. It is not clear whether the outcomes reported were considered most important from the beginning of the intervention or if any success found was reported and associated with the intervention. Only one paper (WHSRO) described other capacity development activities taking place simultaneously within their institution and noted that the outcomes described in their article could have been a result of any of the interventions i.e. they discussed that their intervention might have contributed to the outcomes rather than attributing all the outcomes to their interventions.

In contrast to the many reports of capacity building of individuals, where broadly similar outcomes are used, the 14 included reports of institutional capacity building reported a variety of indicators. We attempted to categorise these outcomes ( Table 3), but this was only our interpretation of what had been reported, limiting comparison between the interventions. It may be that the variety of reported outcomes reflects the exploratory and complex nature of the interventions and that some outcomes are difficult to measure. Other authors have reported similar difficulties in assessing such interventions ^3, 35, 36^.

If common definitions and terminology had been used, the interpretation of the included reports would be more meaningful ^37^. Agreed indicators of inputs, outputs and outcomes relevant to capacity building would enable systematic comparisons to be made. The absence of common terminology for reporting these interventions reflects the lack of a common lexicon around institutional change. This, despite such changes being a major concern of funders and implementers. The development of a common framework for reporting research capacity strengthening interventions would be valuable in enabling systematic reporting and comparison to be made in future. Such a framework has been suggested before ^34^.

For interventions to have a lasting impact on research capacity, the changes made must be sustainable over the long term. Our review highlights three factors that seem important in sustaining research capacity development. Equitable partnerships, with local leadership, were related to sustainability. The importance of local leadership as a driver of improved research capacity in LMICs has been underscored by others ^35, 38^. The authors describing the MEPI efforts in Mozambique compared MEPI to other funding schemes and noted that local leadership determining the priorities for the intervention underpinned success ^39^. This confirms a recommendation from a rapid evidence assessment of 227 studies on research capacity strengthening in LMICs ^3^.

Our findings also suggest that national and local higher education policies are an important factor in stimulating and supporting change. In one paper, the authors commented that to be sustainable, the intervention would require ongoing strong leadership and continued financial support [WHSRO]. Previous researchers support this assertion and have argued that when the higher education system in a country incentivises research, this is likely to be embraced by institutions ^3^.

The majority of the initiatives included in this review relied to some extent on funding from Northern partners. Over many years, Northern partners have made considerable investments in research capacity building in Africa ^40^. However, documenting long term sustainable change has proved challenging. The authors of one of the papers we included reflected on how important an appropriate level of external funding had been as a driver of success [IDIMak]. Local support for African higher education is erratic ^41^ and sustainable improvements in university research capacity will likely require reliable and sufficient external funding for some considerable time.

We undertook this review looking for examples of successful interventions that might inform the development of the CARTA institutionalisation framework. However, this review has not provided any further understanding of how to tailor our institutionalisation programme beyond what we originally agreed. The theory of change underpinning CARTA and further research documenting whether CARTA activities have resulted in institutionalisation may add to a common lexicon and outcome measures ^42^.

## Data availability

### Underlying data

All data underlying the results are available as part of the article and no additional source data are required.

### Extended data

Open Science Framework: Data From: Institutionalising research capacity strengthening in LMICs. https://doi.org/10.17605/OSF.IO/TNPBV ^6^.

This project contains the following extended data:

- Supplementary_Table_1_Search_Strategy_example.docx
- Supplementary_Table_2_Included_studies.docx
- Supplementary_Table_3_Activities_and_outcomes.docx

Extended data are also available on Wits Institutional Repository environment on DSpace (WIReDSpace). http://wiredspace.wits.ac.za/jspui/handle/10539/29290.

### Reporting guidelines

PRISMA checklist for ‘Institutionalising research capacity strengthening in LMICs: A systematic review and meta-synthesis’ https://doi.org/10.17605/OSF.IO/TNPBV ^6^.

Data are available under the terms of the Creative Commons Attribution 4.0 International (CC BY 4.0).
