# Supplementary material for: Institutionalizing research capacity strengthening in LMICs: A systematic review and meta-synthesis
Source: AAS Open Res. 2021 Feb 12;3:43. Originally published 2020 Sep 9. [Version 3] doi: 10.12688/aasopenres.13116.3 (PMC7653640; doi:10.12688/aasopenres.13116.3)
Supplement: Peer review history

# Peer review: Reviewer response for version 1

**DOI:** 10.21956/aasopenres.14216.r28017

**Published:** 2020-11-09

**Authors:** Carla Carrilho

**Affiliations:** 1 Department of Pathology, Maputo Central Hospital, Maputo, Mozambique; 2 Department of Pathology, Faculty of Medicine, Eduardo Mondlane University, Maputo, Mozambique

In the present manuscript the authors performed a very interesting systematic review aiming to identify effective strategies for research capacity building at institutional level in low- and middle-income countries (LMICs), and consequently the long-term sustainability of these actions.   

 The authors describe the initiatives, strategies and outcomes of research capacity reported in different papers that were included in this analysis. They conclude that there is not a systematic way of reporting the results, and the differences in the methodologies and approaches make impossible its comparison.  

 In our opinion, the absence of a standardized way to present the results in the papers analysed, is not a meaning of failure of the implemented projects or initiatives. If it is not well-defined at the beginning, we cannot expect that the report of the results should be presented in a comparable and systematic way.

 The methodology of the work is well done and a clear description of the results is presented. The tables are illustrative of what was found and supplementary material was provided to complement the work. A simple and clear discussion is presented.  

 A lesson to take for the future, that I think is the main conclusion of this paper, is the need to create a conceptual framework to report the positive and negative results of these projects/initiatives, allowing the analyses as well as the comparability among them.

 I recommend the acceptance of this paper for publication, as it brings a very important contribution to report significance of future projects.

Are the rationale for, and objectives of, the Systematic Review clearly stated?

Yes

Is the statistical analysis and its interpretation appropriate?

Yes

Are sufficient details of the methods and analysis provided to allow replication by others?

Yes

Are the conclusions drawn adequately supported by the results presented in the review?

Yes

Reviewer Expertise:

Pathology; cancer

I confirm that I have read this submission and believe that I have an appropriate level of expertise to confirm that it is of an acceptable scientific standard.

# Response

**Published:** 2021-01-28

**Authors:** Ojo Agunbiade

**Affiliations:** Obafemi Awolowo University, Nigeria

We appreciate the author’s commendation on this paper. We will like to mention that we have added an active link to the OSF. There are no changes to the extra files uploaded earlier. Lastly, a few typing errors that were initially unobserved have been addressed in the revised version.

# Peer review: Reviewer response for version 1

**DOI:** 10.21956/aasopenres.14216.r27875

**Published:** 2020-10-06

**Authors:** Emilia Noormahomed

**Affiliations:** 1 Department of Medicine, Division of Infectious Diseases and Global Public Health, University of California, San Diego, San Diego, CA, USA

Although African continent bears a huge burden of disease, it is lacking of enough workforce to provide universal health care and conduct research on the most pressing health issues.

 This is because in LMICs most training and research institutions have limited financial resources and research infrastructure including human resources with research and mentorship skills.

 This systematic review calls the attention of the reader that some past and ongoing initiatives aiming to develop research capacity in LMICs have used different approaches making it difficult to compare amongst them. Some of this research capacity building initiatives have resulted in a sustainable research capacity development, while others did not have a temporal horizon as were targeted to achieving specific goals within a certain period of time. 

 Based on this systematic review, results and analysis done by the authors have concluded that   although the described research capacity initiatives have used different approaches that resulted in different outcomes, they cannot be compared to each other because there was not uniformity. Further, most of them did not use the conceptual framework to develop the capacity building approach in LMICs.

 In addition, in all analysed papers none of the authors have pointed out failures related to the approaches they used only success. This implies that future papers aiming to document results of these initiatives should point out failures, so that it can help implementers to better design future research strategies for capacity building. As such it would be interesting if we could have different counties using the same approach (conceptual framework to develop the capacity building) and compare the results.

 In terms of format, the authors have addressed PRISMA guidelines and provided a comprehensive explanation as well as provided tables with enough information so that similar manuscripts can be replicated elsewhere.

 The discussion is well conducted and has also pointed out the limitations which are related to the different methodologies used from the different authors that were included in the analysis.

 The conclusions are well summarized and pointed out some special features that were considered important to the success of these initiatives namely: a) Equitable partnership and empowerment of local researchers as an important factor that contributed to ensure sustainability, as many initiatives depend on external - northern partners for financing; b) national and local higher education policies to be reviewed whenever appropriated as they are an important factor in stimulating and supporting changes whenever necessary.

 The paper pointed out also some limitations like the non-systematic manner the results were reported, making it impossible to draw comparisons. In addition to that the outcomes that were reported without connection to the inputs, and as such it was difficult to identify any direct contribution of these initiatives.

 In view of the important contributions this paper brings for future initiatives, I strongly recommend that this manuscript should be accepted for indexing.

Are the rationale for, and objectives of, the Systematic Review clearly stated?

Yes

Is the statistical analysis and its interpretation appropriate?

Yes

Are sufficient details of the methods and analysis provided to allow replication by others?

Yes

Are the conclusions drawn adequately supported by the results presented in the review?

Yes

Reviewer Expertise:

Neglected Tropical Diseases: epidemiology, clinical features and development of diagnostic tools. Innovative strategies to train biomedical researchers in LMICs. Health Professionals training.

I confirm that I have read this submission and believe that I have an appropriate level of expertise to confirm that it is of an acceptable scientific standard.
